# Supplementary material for: The perceived barriers and facilitators for chiropractic care in older adults with low back pain; insights from a qualitative exploration in a dutch context
Source: PLoS One. 2023 Apr 12;18(4):e0283661. doi: 10.1371/journal.pone.0283661 (PMC10096496; doi:10.1371/journal.pone.0283661)
Supplement: S1 Appendix — (DOCX) [file pone.0283661.s001.docx]

**Topic guide chiropractic patients**

Hello ….,

My name is…, we have spoken on the phone not long ago. First of all, I would like to thank you for participating in this study. We have recently sent you an information letter and an informed consent form. Did you have any more questions about it? If you have any questions at any moment, please feel free to ask.

First I would like to ask you to tell me a little bit more about yourself. Who are you? What is your age? What do you or did you do for a living? What does your family look like?

**The experienced back pain:**Then let’s dig a little deeper into the back pain now.

- Can you tell me more about your back pain?
- How long have you been experiencing these back problems?
- Have you had back pain before this episode?
  - If so: what did you do then to get rid of your back pain?
- How has your back pain changed your work or activities of daily life?
- What is, according to you, the cause of the back pain? What is going on?
  - Do you find it very important to learn more about this? Where do you think you could find this information? What other information would you like to have about your back?

**Pathway to care:**For our research, it is important to know what you did in the past to seek care for your low back pain. Therefore the next questions will be concerned with the care you have received up till now.

- Did you seek help from a doctor or therapist for these complaints?
  - If so: How did you experience this care?
  - If so: Is there anything you have missed with the previous treatments from therapists or specialists?
  - If so: Do you feel like you have received sufficient information from the care provider?
  - If so: Did you see anyone else?
  - If not: Have you tried any methods to relieve the pain or optimize the functionality of your back?
  - What has had the best effect up till now?
- How did you end up at the chiropractor? Did another therapist recommend it? Did you gain information from the internet or have you been recommended by family or acquaintances?
  - If recommended: Did you search for more information on the internet?
  - If recommended: Did you get recommended any other treatments or therapies?
- Have relatives, family, or doctors/therapists ever recommended you to go see a therapist or specialist?
  - If so: what did you do with this advice?
  - If so: Have you googled this therapy or specialist?
- Have you seen your GP for low back pain?
  - If not: why not?
  - If so: What did the GP say?
  - If so: How did you experience this?
  - If so: Did your GP refer you? If so, to whom did he/she refer you?
- Have you ever considered trying any other therapies or treatments but decided not to undergo it?
  - If so: which?
  - If so: Why have you never tried?
  - If so: why did you choose chiropractic care over other treatments? (eg. Osteopathy or acupuncture)
  - If not: Why have you never considered another treatment or therapy?

**The Chiropractor**

Now there will be some specific questions on your care seeking pathway for the chiropractor.

- Why have you made an appointment with the chiropractor NOW specifically?
- Did you have any doubts before making an appointment with the chiropractor?
  - Did these doubts change when you made an appointment with the chiropractor?
- How did you experience the process of appointment making?
  - Were there any hurdles when making the appointment?
  - Was it hard or easy to find a chiropractor?
  - How long did it take to get an appointment?
- What stimulated you to make an appointment with the chiropractor?
- What do you know about chiropractic care?
- What drew you to chiropractic?
- How are treatments covered by your health care insurance?
- What information do you hope to receive from the chiropractor?
  What do you think the chiropractor can mean for you?
- What would you advise chiropractors to make chiropractic care more accessible?

**Is there anything I have forgotten?**

**To close it off:**To close it off, I have some general questions about you:

- What is the gender you identify with?
- What is your age?
- What is your highest level of education?
- What is/was your profession?

**Topic guide non-chiropractic patients**

Hello ….,

My name is…, we have spoken on the phone not long ago. First of all, I would like to thank you for participating in this study. We have recently sent you an information letter and an informed consent form. Did you have any more questions about it? If you have any questions at any moment, please feel free to ask.

First I would like to ask you to tell me a little bit more about yourself. Who are you? What is your age? What do you or did you do for a living? What does your family look like?

**The experienced back pain:**Then let’s dig a little deeper into the back pain now.

- Can you tell me more about your back pain?
- How long have you been experiencing these back problems?
- Have you had back pain before this episode?
  - If so: what did you do then to get rid of your back pain?
- How has your back pain changed your work or activities of daily life?
- What is, according to you, the cause of the back pain? What is going on?
  - Do you find it very important to learn more about this? Where do you think you could find this information? What other information would you like to have about your back?

**Pathway to care:**For our research, it is important to know what you did in the past to seek care for your low back pain. Therefore the next questions will be concerned with the care you have received up till now.

- Did you seek help from a doctor or therapist for these complaints?
  - If so: How did you experience this care?
  - If so: Is there anything you have missed with the previous treatments from therapists or specialists?
  - If so: Did you see anyone else?
  - If not: Have you tried any methods to relieve the pain or optimize the functionality of your back?
  - What has had the best effect up till now?
- Have relatives, family, or doctors/therapists ever recommended you to go see a therapist or specialist?
  - If so: what did you do with this advice?
  - If so: Have you googled this therapy or specialist?
- Have you seen your GP for low back pain?
  - If not: why not?
  - If so: What did the GP say?
  - If so: How did you experience this?
  - If so: Did your GP refer you? If so, to whom did he/she refer you?
- Have you ever considered trying any other therapies or treatments but decided not to undergo it?
  - If so: which?
  - If so: Why have you never tried?
  - If not: Why have you never considered another treatment or therapy?

**The chiropractor**Now we’ll ask some questions specific to chiropractic care.

- Have you ever heard about a chiropractor?
  - What do you know about chiropractic?
- Have you ever been to a chiropractor?
  - If so: What was your experience?
  - If not: why didn’t you go to a chiropractor?
- Why have you never been to a chiropractor?
  - Did you have specific doubts?
  - What do you think could take these doubts away?
- Would you consider going to a chiropractor in the future for your low back pain?
- What would motivate you to go to a chiropractor?
  - How would the coverage from the health care insurance influence this?
  - How would this change if the chiropractor was closer by?
  - Would this change if you could be seen by a chiropractor earlier than by a physiotherapist for example?
  - Does the media have any influence on this? Eg. Television, radio, the newspaper or the internet?
- Where would you expect an advertisement for a chiropractor?
- Where would you expect to find more information on chiropractic care?
- What draws you to chiropractic care, if anything draws you to it?
- Do you know how chiropractic care is covered by health care insurance?
- What would you advise chiropractors to make chiropractic care more accessible?

**Is there anything I have forgotten?**

**To close it off:**To close it off, I have some general questions about you:

- What is the gender you identify with?
- What is your age?
- What is your highest level of education?
- What is/was your profession?

**Topic guide focus group**

Good evening everyone. Thank you so much that you’re willing to help us by sharing your professional view on older adults with low back pain and chiropractic care.

My name is Lobke. Today I am the moderator of this focus group. Sidney and Annemarie are here to make sure everything runs smoothly. With this focus group, we want to gain information from you, as professionals, on the barriers and facilitators that older adults may experience when seeking care for their low back pain, and then specifically chiropractic care.

We have a specific list of questions that we want to go through. First I would like to start with 4 questions, that we can answer and elaborate on together. If you wish to, you can also respond to the answers of the other focus group members. We want this to resemble a natural conversation. After two questions we will have a little break to stretch our legs.

My task in this focus group is to make sure that everyone gets to say what they want, but also to guide the conversation to stay on topic.

To make sure everything runs smoothly and we receive useful information from this focus group, I would like to set some ground rules.

1. Would you please all turn on your webcam and keep the webcam and microphone on at all times? This will make the conversation as natural as possible.
2. We want to be able to hear you clearly. Despite that we do want you to respond to each other, it is important to do this one person at a time.
3. Everyone’s response is important. If you do not agree with someone, we would like to hear it, but please remain respectful to each other.
4. Please make sure that this conversation remains confidential. We want everyone to feel free to share how they feel and be sure that the conversation is not shared with others.
5. We record this focus group to gain information as specifically as possible from this focus group.
6. Please stay focused. If we would be in a room together, having a conversation, you wouldn’t get distracted by your phone or e-mail either.

These are some basic rules, do you all agree or have any more questions on this?

Besides this information, we have already sent you an information letter and an informed consent form before the interview. Have you all received this? Does anyone have any questions on this?

First, I would like to do an introductory round so that we get to know each other a little bit better. In the chat, I shared a link to a Jamboard. On the first page, there is a map of the country. Let’s all go there first. Then I would like everyone to do one by one introduce yourself by stating your name, your profession and your location. Then you can post a sticky note with this information on the location where you are to get a visual overview.

Well, now we know each other a little better, I would like to continue with the questions.

1. What do you all think the facilitating factors are for older adults with low back pain to seek care from a chiropractor?

All answers that are mentioned will be posted on the pinboard with a virtual sticky note on the second page. Then we discuss these topics and cluster them together if they are congruent.

1. What do you think the limiting factors are for older adults with low back pain to seek care from a chiropractor?

All answers mentioned will be posted on the pinboard with a virtual sticky note on the third page. Then we discuss these topics and cluster them together if they are congruent.

Break. During the 5 min. Break, the post-its will be transferred from slide 2 to slide 4 and from slide 3 to slide 5.

1. Now I want to discuss a few factors that were established in the first part of our study, during the interviews. I am curious about what you all think of the established factors. We have asked older adults to establish the barriers and facilitators that they experienced either seeking or not seeking care from a chiropractor. On slide 4 you can see the factors that you have all agreed on before the break and the factors that were identified during the interviews with older adults.
   1. Do you think these factors differ from each other?
   2. The responses that weren’t mentioned during the focus group, but were mentioned by the older adults: what do you think about these factors?
      1. If so: why did you not mention it earlier?
      2. If not: why do you think your opinion differs from that of the interviewed older adults?
   3. The responses mentioned in the focus group but not in the interviews:
      1. Why do you think these factors weren’t mentioned by the older adults?
      2. What do you think about the difference in answers between the interviews and focus group?
2. Now, let’s look at the barriers mentioned in the interviews.
   1. Do you think these factors differ from each other?
   2. The responses that weren’t mentioned during the focus group, but were mentioned by the older adults: what do you think about these factors?
      1. If so: why did you not mention it earlier?
      2. If not: why do you think your opinion differs from that of the interviewed older adults?
   3. The responses mentioned in the focus group but not in the interviews:
      1. Why do you think these factors weren’t mentioned by the older adults?
      2. What do you think about the difference in answers between the interviews and focus group?
3. Is there any other subject that we haven’t touched upon? Anything else you think is important to discuss, any other factors that could influence the care seeking behaviour of older adults with low back pain?
