# Supplementary material for: The perceived barriers and facilitators for chiropractic care in older adults with low back pain; insights from a qualitative exploration in a dutch context
Source: PLoS One. 2023 Apr 12;18(4):e0283661. doi: 10.1371/journal.pone.0283661 (PMC10096496; doi:10.1371/journal.pone.0283661)
Supplement: Attachment — Submitted filename: reactie PlosOne.docx [file pone.0283661.s002.docx]

Amsterdam, The Netherlands

February 7th, 2023

Manuscript: CHMT-D-22-00008

What are the perceived barriers and facilitators for chiropractic care in older adults with low back pain?

Dear Editor-in-Chief,

Thank you for the opportunity to reconsider publication of our manuscript following revision and modification.

Below you will find our response to the comments by the editor and reviewer. The necessary changes have been made to the manuscript and have been uploaded in the system. These changes are clearly indicated in the editorial mode (‘track changes’).

The ‘Role of Funder’ statement is the following:

This study is funded by European Centre for Chiropractic Research Excellence (ECCRE), located in Odense, Denmark (grant number 01-20160NL/MvT) as well as funded by the Nederlandse Chiropractoren Associatie (NCA), located in Emmeloord. The funders had no role in study design, data collection and analysis, decision to publish, or preparation of the manuscript.

The ‘competing interests’ statement is the following:

Four authors (LDLR, ADZ, CM and SMR) are chiropractors who work in clinical practice, but have no direct financial interests linked with this survey. This does not alter our adherence to PLOS ONE policies on sharing data and materials.

The ‘data availability’ statement is the following:

All relevant data are within the manuscript and its supporting information files. The transcripts of the interviews and focus group cannot be shared publicly due to privacy reasons as participants can be identified. This is in accordance with European Data Protection Regulation (GDPR) (<https://libguides.vu.nl/rdm/gdpr-privacy>). Individual participants’ data are available upon request from the principal investigator (Sidney Rubinstein, [s.m.rubinstein@vu.nl](mailto:s.m.rubinstein@vu.nl)) for researchers who meet the criteria for access to confidential data).

We look forward to your reaction. On behalf of all authors sincerely,

Lobke De la Ruelle

Correspondence:

Lobke De la Ruelle

Department Health Sciences,

Faculty of Science,

Vrije Universiteit Amsterdam,

Van der Boechorststraat 7

1081 BT Amsterdam,

The Netherlands

l.p.delaruelle@vu.nl

**Response to the reviewers**

**Article title:** What are the perceived barriers and facilitators for chiropractic care in older adults with low back pain?

We would like to thank the reviewer for her valuable comments and suggestions for improvement. Below we address each of the specific comments in detail.

| **Editor** |  |  |
| --- | --- | --- |
| 1. *comment editor:* We note that the grant information you provided in the ‘Funding Information’ and ‘Financial Disclosure’ sections do not match. When you resubmit, please ensure that you provide the correct grant numbers for the awards you received for your study in the ‘Funding Information’ section. | *Response:* At this moment I cannot find the ‘financial disclosure’. In the ‘Funding information’, We have changed the data to the following: | P25-26 lines 697-701  This study is funded by European Centre for Chiropractic Research Excellence (ECCRE), located in Odense, Denmark (grant number 01-20160NL/MvT) as well as funded by the Nederlandse Chiropractoren Associatie (NCA), located in Emmeloord. The funders had no role in study design, data collection and analysis, decision to publish, or preparation of the manuscript. |
| *Comment editor:* Thank you for stating the following financial disclosure:  "This study is funded by European Centre for Chiropractic Research Excellence (ECCRE), located in Odense, Denmark (grant number 01-20160NL/MvT) as well as funded by the Nederlandse Chiropractoren Associatie (NCA), located in Emmeloord. "  Please state what role the funders took in the study.  If the funders had no role, please state: "The funders had no role in study design, data collection and analysis, decision to publish, or preparation of the manuscript."  If this statement is not correct you must amend it as needed.  Please include this amended Role of Funder statement in your cover letter; we will change the online submission form on your behalf. | *Response*:  This has been adapted | See 1 |
| *Comment editor*: Thank you for stating the following in the Funding Section of your manuscript:  "This study is funded by European Centre for Chiropractic Research Excellence (ECCRE), located in Odense, Denmark (grant number 01-20160NL/MvT) as well as funded by the Nederlandse Chiropractoren Associatie (NCA), located in Emmeloord. "  We note that you have provided funding information that is not currently declared in your Funding Statement. However, funding information should not appear in the Acknowledgments section or other areas of your manuscript. We will only publish funding information present in the Funding Statement section of the online submission form.  Please remove any funding-related text from the manuscript and let us know how you would like to update your Funding Statement. Currently, your Funding Statement reads as follows:  "This study is funded by European Centre for Chiropractic Research Excellence (ECCRE), located in Odense, Denmark (grant number 01-20160NL/MvT) as well as funded by the Nederlandse Chiropractoren Associatie (NCA), located in Emmeloord. "  Please include your amended statements within your cover letter; we will change the online submission form on your behalf. | *Response*:  See 1 | See 1 |
| *Comment editor:* Thank you for stating the following in the Competing Interests section:  "Four authors (LDLR, ADZ, CM and SMR) are chiropractors who work in clinical practice, but have no direct financial interests linked with this survey."  Please confirm that this does not alter your adherence to all PLOS ONE policies on sharing data and materials, by including the following statement: "This does not alter our adherence to  PLOS ONE policies on sharing data and materials.” (as detailed online in our guide for authors http://journals.plos.org/plosone/s/competing-interests).  If there are restrictions on sharing of data and/or materials, please state these. Please note that we cannot proceed with consideration of your article until this information has been declared.  Please include your updated Competing Interests statement in your cover letter; we will change the online submission form on your behalf. | *Response:* This has been adapted as followed: | P 25, lines 693-696  ***Competing interests***  Four authors (LDLR, ADZ, CM and SMR) are chiropractors who work in clinical practice, but have no direct financial interests linked with this survey. This does not alter our adherence to PLOS ONE policies on sharing data and materials. |
| *Comment editor:* In your Data Availability statement, you have not specified where the minimal data set underlying the results described in your manuscript can be found. PLOS defines a study's minimal data set as the underlying data used to reach the conclusions drawn in the manuscript and any additional data required to replicate the reported study findings in their entirety. All PLOS journals require that the minimal data set be made fully available. For more information about our data policy, please see http://journals.plos.org/plosone/s/data-availability.  Upon re-submitting your revised manuscript, please upload your study’s minimal underlying data set as either Supporting Information files or to a stable, public repository and include the relevant URLs, DOIs, or accession numbers within your revised cover letter. For a list of acceptable repositories, please see http://journals.plos.org/plosone/s/data-availability#loc-recommended-repositories. Any potentially identifying patient information must be fully anonymized.  Important: If there are ethical or legal restrictions to sharing your data publicly, please explain these restrictions in detail. Please see our guidelines for more information on what we consider unacceptable restrictions to publicly sharing data: http://journals.plos.org/plosone/s/data-availability#loc-unacceptable-data-access-restrictions. Note that it is not acceptable for the authors to be the sole named individuals responsible for ensuring data access.  We will update your Data Availability statement to reflect the information you provide in your cover letter. | *Response:* The transcripts of the interviews and focusgroup cannot be shared publicly because of privacy reasons. | **P25, line 686-692**  ***Availability of data and materials***  All relevant data are within the manuscript and its supporting information files. The transcripts of the interviews and focus group cannot be shared publicly due to privacy reasons as participants can be identified. This is in accordance with European Data Protection Regulation (GDPR) (<https://libguides.vu.nl/rdm/gdpr-privacy>). Individual participants’ data are available upon request from the principal investigator (Sidney Rubinstein, [s.m.rubinstein@vu.nl](mailto:s.m.rubinstein@vu.nl)) for researchers who meet the criteria for access to confidential data). |
| *Comment editor:* Please upload a new copy of Figure 2 as the detail is not clear. Please follow the link for more information: https://blogs.plos.org/plos/2019/06/looking-good-tips-for-creating-your-plos-figures-graphics/" https://blogs.plos.org/plos/2019/06/looking-good-tips-for-creating-your-plos-figures-graphics/  [Note: HTML markup is below. Please do not edit.] | *Response:* The text size has been adapted in some of the bubbles, making the image much clearer. |  |

| **Reviewer** |  |  |
| --- | --- | --- |
| 2. *comment reviewer:* This study is a good early work by a young investigator, exploring the perspectives of Dutch older adults about seeking chiropractic care. The background would be strengthened by inclusion of more detail about role of chiropractic in Dutch healthcare system - funding, number of chiropractors, training - this would serve to educate readers about the profession in this context - the lack of which info was mentioned as a barrier by patients and providers alike. | *Response:* Thank you so much for your kind and encouraging words.  When it comes to the role of chiropractors in the health care system in the NL, I think it is indeed a good idea to give some more information for context as it surely differs from one country to the next. | P 5, lines 106-111  Added: In the Netherlands, chiropractic care is considered ‘alternative care’. For the Dutch LBP patients this means that health care insurance only covers some, but never 100% of the costs. The profession is relatively small with only 299 practitioners in a country with more than 17 million inhabitants. Despite their small numbers, in 2019 more than 1 million treatments were delivered by chiropractors (unpublished observations) in the Netherlands. |
| 3. *Comment reviewer*: Lines 90-95 - References 2-4 seem to have dropped off, making credibility of statements difficult to ascertain. | *Response*:  There appears to have been an electronic glitch. My apologies. | p. 27-28 lines 620-238. This has become references 4-6. |
| 4. *Comment reviewer*: Lines 103-104 - Qualitative study healthcare seeking back pain older adults - There's an extensive qualitative literature on this topic, enough that there's a meta-synthesis and systematic review on care seeking for LBP. You might not find it by looking for 'barriers and facilitators' alone, but it's there - AND there's info about this in regards to chiropractic care, often in the context of interprofessional care (MD/DC or DC/PT) - as well as disabling pain (Makris et al). This reviewer is very familiar with this work, and encourages the team to look more closely at this literature to inform both the background and the discussion of this paper (Essential revision). | *Response*:  This is a good point. We were aware of this review, but felt that it was not relevant because it did not examine chiropractic care specifically, care seeking behaviour nor examine the patient’s perspective. Nevertheless, upon further consideration, feel that it provides important context, Therefore have added the following information: | P5, lines 120-124  Older adults are, however, less likely to seek care for their LBP due to the assumption that LBP is a normal part of ageing. Moreover, older individuals tend to display a negative attitude towards pharmacological and/or surgical interventions and deem their LBP less important than other comorbidities (7, 8).  P 22, lines 479-485  Other qualitative studies examined the perspectives of older adults on LBP and on the care they received from the chiropractor(2, 8). One of the participants (8) stated they consider chiropractic care a primary treatment modality for LBP. However, the role of chiropractic care in the health care system can differ greatly between countries Our study demonstrates lack of awareness on chiropractic care in the Netherlands. This would suggest that the results of studies, conducted in other countries and settings are not generalizable, and are context specific. |
| *Comment reviewer*: Lines 134-137 - Design. No references are provided for the numerous methodologies mentioned: exploratory qualitative, case study, naturalistic design, constructivist lens, factor(analysis?), and lived experience (typically used to describe phenomenology - which is not in alignment with a constructivist lens). Pick a design (exploratory/descriptive qualitative is sufficient), include references that guided this approach, and include sufficient detail that another qualitative researcher can tell what was done and how this approach informed the thinking/data analysis. (Essential revision) Move COREQ statement up here, too, to allow reader to know how presentation is being structured. | *Response:* The COREQ statement has been moved to ‘design’.  Design has been adapted to: | Page 8: line 161-167  We constructed an explorative, qualitative case study, with the case defined as old individuals engaged in the process of care seeking for a lower back pain-related problem (13). This approach was deemed appropriate to develop a thick description of individual and shared experiences occurring during this process; both individuals who had chosen chiropractic care and those who did not. The 32-item checklist for interviews and focus groups [Consolidated criteria for reporting qualitative research (COREQ)] was used as a guideline for the report ((14)). |
| 5. *Comment reviewer*: Lines 138-141 - Theoretical approach - thematic analysis in an analytic approach than a theoretical lens. You might have a theoretical approach listed above from a disciplinary lens, which will require more explication and reference here. What would be important to include is some theoretical lens through which you viewed old age - disengagement, continuity, activity theories? Integrity vs despair? Others? HOW the research team view old people and the aging process will deeply influence how questions were asked, and the themes discovered- for example, 55 year old people are considered old rather than midlife for inclusion purposes...this is a lens through which aging is viewed. This is an essential revision that must be addressed. | *Response:* The theoretical approach paragraph has been replaced by the following: | Page 8: line 171-181 From a theoretical perspective, we drew from a recent comprehensive literature review conducted by a Dutch research group (15). In their investigation, Hupkens et al. highlight several processes focused on maintaining a high quality of life, living independently and being to engage and participate in society as integral to attaining meaning in life in older age. Our position, although perhaps tacit, is that LBP represents a factor that has the potential to rob older adults therefore of their valued needs if their care seeking is unsuccessful.  Our definition of what age might define an older person was pragmatic in nature, and was aimed at alignining with current published literature from within the discipline of chiropractic. In this regard 55 years of age is considered ‘older’ (16).  Our study was also underpinned by a constructivist stance, as we sought to create a meaningful co-construction of pathways to careseeking through the experiences of participant stakeholders. (13) |
| 6. *Comment reviewer:* Lines 145 - Include any pertinent references to the BACE-C study, as well as some key eligibility criteria to larger study. | *Response:* The BACE-C has not been published yet, but I have referenced the published protocol. | Reference 16. Jenks AD, Hoekstra T, Axén I, de Luca K, Field J, Newell D, et al. BAck complaints in the elders - chiropractic (BACE-C): protocol of an international cohort study of older adults with low back pain seeking chiropractic care. Chiropractic & Manual Therapies. 2020;28(1):17. |
| 7. *Comment reviewer:* Lines 146-149 - Consider how these exclusions might have biased your findings in the discussion. For example, the decision to exclude patients who have received previous chiropractic care does not UNBIAS this study - it just differently biases the study, because people who had NOT received chiropractic care for at least 55 years have STRONG OPINIONS about chiropractic care - it kept them off the table! This decision is a limitation, because we now don't know what is a barrier or facilitator for people who are positively inclined to use chiropractic - so cannot build on what works! | *Response:* The reviewer raises an important point. We added a paragraph in the discussion dedicated to this topic.  Also we have adapted our aim to make it more clear that we are only looking at patients without prior experience. | P 3, lines 51-53  The aim of this paper was to explore the experiences of older adults with LBP, seeking chiropractic care for the first time, in order to identify perceived barriers and facilitators in this process.  P 23, lines 501-503 Patients with prior experience with chiropractic care were excluded because those with a prior (positive) bias are likely to view the abarriers and facilitators to chiropractic care differently than those who are naïve to care. |
| 8. *Comment reviewer :* Lines 159 - Tech reference for Zoom company (as for any scientific/tech company) and rationale as to why this technology was used over other similar technologies. | *Response*: The reference has been added. The following text has been added on the rationale.  Zoom was chosen as software as this was the software currently used at the Vrije Universteit Amsterdam | P24, lines 537-542  The decision to conduct the interviews over video call was influenced by the COVID-19 pandemic(23, 24). To protect the more fragile participants, we chose to conduct video calls rather than live interviews. This choice, however, potentially led to selection bias, because some participants were excluded if they did not have access to a computer. However, we noticed that even some of the digitally challenged managed to participate with the help of their family. Therefore, we are of the opinion that this bias was limited. |
| 9. *Comment reviewer*: Line 176 - How were interviews transcribed - by the Zoom software, or by the investigators? Provide reference to support this decision. How was translation from Dutch to English handled for publication? This should be in methods not results and include the process and who completed this task. | *Response*: The information has been added. | P. 11 lines 235-239  All interviews were conducted in Dutch via online video call (Zoom), were voice recorded, and transcribed verbatim. The transcriptions were generated by the investigators, chiropractic colleagues or by a transcription company. Solely the quotes used in this article were translated to English by a person fluent in both languages. |
| 10. *Comment reviewer*: Data collection - no reference are provided for decisions make in stage 1 or 2. Methods need references. | *Response:*  The reference has been added.  Also a paragraph has been added at the bottom of ‘data collection’. | Reference 17. Green J, Thorogood N. Qualitative Methods for Health Research: SAGE Publications; 2013.  P. 12 lines 266-270  Our strategy in phase 1 and 2 was aimed at optimizing data completeness(19). Specifically, interviews allowed for a thick description of individual experiences, whilst focus groups captured a range of barriers and facilitators experienced in the responder group which accentuated differences and similarities. This methodological strategy is thought to optimize qualitative-qualitative triangulation, which comprises the combination of different qualitative data collection methods. |
| 11. *Comment reviewer*: Interviews - how was oversight provided for this first time interviewer? Which team member handled this training and oversight? | *Response*: A few lines have been added to the text to describe this. | P11 lines 226-231  The interviewer followed a course ‘qualitative research in the field of health care’ with six hours of lectures and nine hours of practical workgroups. After four initial interviews, transcripts were evaluated by the project group and adaptations made to the interview guide. Four questions were added to aid the participants in answering questions with greater reflective depth, whilst still keeping the interviews well bracketed. Technical elements of the interview process were also discussed in order to assist the researcher in developing her interviewing style. |
| 12. *Comment reviewer*: Line 180 - Purposive sampling needs reference. And more description as to how this happened. | *Response*: Reference and following information has been added: | Reference 17. Green J, Thorogood N. Qualitative Methods for Health Research: SAGE Publications; 2013  P. 9 lines 195-197  To achieve this, potential participants were contacted if they balanced these factors out. |
| 13. *Comment reviewer*: Line 199 - MaxQDA needs company details. How was intercoding agreement assessed? | *Response:* Reference has been added. Text on intercoder agreement has been adapted | P13 lines 273-277  The first 4 interviews with participants seeking care and the first 2 interviews with participants not seeking care were independently coded by two investigators (LPR and ADZ) using open coding and later discussed between them until mutual agreement to increase intercoder agreement. |
| 14. *Comment reviewer:* Line 201 - Member check needs reference. | *Response:* Reference has been added | Reference 17. Green J, Thorogood N. Qualitative Methods for Health Research: SAGE Publications; 2013 |
| 15. *Comment reviewer:* Data analysis - more detail on thematic analysis are needed as it's unclear who did what and how codes/themes were generated (particularly given the many theoretical/methodological frames mentioned). | *Response:*  We have adapted the following: | P. 13. Lines 286-292 A thematic analysis was performed. This process involved codifying and then abstracting connected data to common themes. Initially, line-by-line inductive coding was performed to define and develop a codebook. Two study collaborators acted as code checkers, providing critical feedback and subsequent refinement of the code book. The code book was then used to code subsequent interviews deductively. |
| 16. *Comment reviewer:* Data analysis - there's no mention about how participants were managed as "cases" to understand individual patterns before group patterns - this means this study isn't a case study. So that's one design it will be easy to let go of in the revision! | *Response:* That is true. The methodology was incorrect. |  |
| 17. *Comment reviewer:*   Figure 1 - Need more information to interpret this figure, including details that this is about care seeking! It's unclear why words are in different sizes, what the various arrows mean. Specific discussion about why chiropractors did not engage in any referral would be helpful. The figure is also messy from a 'time' perspective - it's unclear if this is 1 patient who did all this (a case) or a general sum up, but it looks like GPs are late in the game (reading figure for 'time' from left to right), but they probably come first. Make more meaning with figure and describe better in text. Figure legends are also needed. Lines 248 - this info about how patients found chiropractor could be included in figure to better explicate healthcare seeking (walked past clinic, flyer, etc). | *Response:* The purpose of the figure is to illustrate the different healthcare pathways the participants have taken. We have added more description. The text size has been adapted, and the thickness of the lines has been modified and described underneath the figure. | P 15, lines 333-335  Figure 1 demonstrates the different pathways to healthcare providers the participants had followed up to the moment of the interview.  Figure 1 The pathways the participants of this study took up to the interviews. The thickness of the arrows indicates the proportion of participants that followed this pathway. |
| 18. *Comment reviewer:* Table 1 - Inclusion of detail on which providers had received care from would offer additional info to make Figure 1 and 2 clearer. | *Response:* Additional information would make the table challenging to read, as every patient has followed different pathways. We trust that the point is made given the additional explanation above. |  |
| 19. *Comment reviewer:* Figure 2 - This figure is also very messy and almost unreadable - formatting per journal instructions are needed. The 4 primary themes/factors are floating in space - link these 4 to a central theme, which is 'care seeking behavior for LBP'. From there, there's no legend that tells the reader how to read the figure. What's a circle, hexagon, or rectangle mean? Are there NO links between the 4 main themes? What's a barrier? What's a facilitator? Make figure clear on all levels. Are some themes from patients and other from focus groups with providers? What's the distinction? Are some from the chiropractic patients - or those that did not choose the chiropractor? | *Response:*  More information has been provided in the results section where the figure is presented as well as in the legend. | P 16, lines 349-353 The barriers and facilitators are found in every theme and most subthemes were mentioned both as a barrier and as a facilitators, such as: the awareness, effectiveness of other therapies and financial reimbursement. For example: if someone experienced enough benefit from treatment A, they will not seek for treatment B. While people that did not have enough pain relief with treatment A, will continue seeking for treatment B.  Figure 2  The four identified themes of older adults seeking chiropractic care for their LBP. The dark circles represent the themes, the hexagons represent the subthemes and the rounded rectangles represent the code families. |
| 20. *Comment reviewer:* Reconsider "generic" as a theme and consider "patient-centered" or "patient factors". These were about patients' lives and should be honored as such*.* | *Response:* We discussed the name of this theme, but do not feel this theme encompasses patient-centred factors. These factors are not clinic-specific nor therapy-specific. For example, distance, waiting time, ease of making an appointment and effectiveness of other therapies will always determine whether someone is interested in trying something new. The distance, waiting time and ease of making an appointment might not even be specific to healthcare alone, but can apply to choosing a school for your child or a photography course or the restaurant for your celebratory dinner. Therefore I think generic is rather fitting. | This has not been modified. |
| 21. *Comment reviewer:* Discussion - more references needed throughout. | *Response:* References added in the added sectons | Reference 2 Makris UE, Melhado T, Lee SC, Hamann HA, Walke LM, Gill TM, et al. Illness representations of restricting back pain: the older Person’s perspective. Pain Med. 2014;15(6):938-46.  Reference 8 Lyons KJ, Salsbury SA, Hondras MA, Jones ME, Andresen AA, Goertz CM. Perspectives of older adults on co-management of low back pain by doctors of chiropractic and family medicine physicians: a focus group study. BMC Complement Altern Med. 2013;13:225.  Reference 23 Archibald MM, Ambagtsheer RC, Casey MG, Lawless M. Using Zoom Videoconferencing for Qualitative Data Collection: Perceptions and Experiences of Researchers and Participants. International Journal of Qualitative Methods. 2019;18:1609406919874596.  Reference 24 Lobe B, Morgan D, Hoffman KA. Qualitative Data Collection in an Era of Social Distancing. International Journal of Qualitative Methods. 2020;19:1609406920937875. |
| 22. *Comment reviewer:* Availability of data statement is not same in manuscript as in cover | *Response:*  This has been adapted. | All relevant data are within the manuscript and its supporting information files. The transcripts of the interviews and focus group cannot be shared publicly due to privacy reasons as participants can be identified. This is in accordance with European Data Protection Regulation (GDPR) (<https://libguides.vu.nl/rdm/gdpr-privacy>). Individual participants’ data are available upon request from the principal investigator (Sidney Rubinstein, [s.m.rubinstein@vu.nl](mailto:s.m.rubinstein@vu.nl)) for researchers who meet the criteria for access to confidential data). |
| 23. *Comment reviewer:* Did the lead author not take part in conceptualization of study? | *Response:* That is correct |  |
| 24. *Comment reviewer:* Requires copyediting for English language; non-standard referencing for journal. | *Response:* The English language has been checked by a native English speaker. Adaptations have been made throughout the text and changes have been tracked. |  |
